# A Comparative Study between Onion Peel Extracts, Free and Complexed with β-Cyclodextrin, as a Natural UV Filter to Cosmetic Formulations

**DOI:** 10.3390/ijms242115854

**Published:** 2023-11-01

**Authors:** Mariana A. Messias, Sara M. Ferreira, Loleny Tavares, Lúcia Santos

**Affiliations:** 1 LEPABE—Laboratory for Process Engineering, Environment, Biotechnology and Energy, Faculty de Engineering, University of Porto, Rua Dr. Roberto Frias, 4200-465 Porto, Portugal; mariana.aranha.messias@gmail.com (M.A.M.); up201604659@edu.fe.up.pt (S.M.F.); 2 ALiCE—Associate Laboratory in Chemical Engineering, Faculty de Engineering, University of Porto, Rua Dr. Roberto Frias, 4200-465 Porto, Portugal; 3 ESAN—School of Design, Management and Production Technologies Northern Aveiro, University of Aveiro, Estrada do Cercal 449, Santiago de Riba-UI, 3720-509 Oliveira de Azeméis, Portugal; tavaresloleny@ua.pt; 4 CICECO-Aveiro—Institute of Materials, Campus Universitário de Santiago, University of Aveiro, 3810-193 Aveiro, Portugal; 5 EMaRT Group—Emerging: Materials, Research, Technology, School of Design, Management and Production Technologies Northern Aveiro, University of Aveiro, Estrada do Cercal, 449, 3720-509 Oliveira de Azeméis, Portugal

**Keywords:** sunscreen, onion peel, phenolic compounds, UV filters, microencapsulation

## Abstract

The growing concern regarding the adverse effects of synthetic UV filters found in sunscreens has spurred significant attention due to their potential harm to aquatic ecosystems and human health. To address this, the present study aimed to extract and microencapsulate sensitive bioactive compounds derived from by-product onion peel (OP) by molecular inclusion using β-cyclodextrin as the wall material. Identification and quantification of bioactive compounds within the extract were conducted through high-performance liquid chromatography (HPLC-DAD) analysis, revealing quercetin and resveratrol as the primary constituents. The photoprotection capacity, evaluated by the sun protection factor (SPF), revealed a protection factor comparable to the value for a synthetic UV filter. The produced microparticles presented high antioxidant capacity, significant photoprotection capacity, encapsulation efficiency of 91.8%, mean diameter of 31 μm, and polydispersity of 2.09. Furthermore, to comprehensively evaluate the performance of OP extract and its potential as a natural UV filter, five O/W emulsions were produced. Results demonstrated that microparticles displayed superior ability in maintaining SPF values over a five-week period. Photoprotection evaluation–skin reactivity tests revealed that both extract and microparticles absorb UV radiation in other regions of UV radiation, revealing their potential to be used as a natural UV filter to produce a sustainable and eco-friendly value-added sunscreen.

## 1. Introduction

The skin holds the distinction of being the largest organ in the human body, constituting approximately 16% of the total body weight [1]. It acts as the first barrier between the human body and external factors, such as radiation. Furthermore, it also plays a crucial role in a person’s physical appearance, which is the reason behind the interest in keeping it healthy [2,3].

Sunlight exposure is essential for the human body to produce vitamin D and induction of β-endorphin expression. As a result, people are advised to spend a minimum of 20 min a day outside. However, unprotected and prolonged sun exposure is harmful to the skin and can cause several dermatological conditions such as solar sunburn, hyperpigmentation, and skin cancer [4,5,6]. Sunscreen application before sunlight exposure maximizes the benefits and minimizes the harm of radiation on the skin. As a results, sunscreens have become one of the most sought and consumed skincare products in the world. Sunscreens are efficient if they contain UV filters to combat both type B and type A UV radiation. There are several synthetic UV filters in the market, which have been studied and approved by different regulatory associations. UV filters are divided in two main categories: inorganic, which reflect UV radiation, and organic, which absorb UV radiation. However, concerns about their safety in the environment have been raised in recent years, such as their noxious effect on marine species, leading to some UV filters being removed from the market [5].

As an alternative to synthetic UV filters, natural compounds rich in antioxidants and with photoprotector capacity [7] have been gaining interest from scientists, such as plant extracts rich in polyphenols [8,9].

Agricultural by-products represent a viable option to explore as a source of bio-compounds. The exponential growth in the world’s population has led to enormous consumption of goods, including foods of agricultural origin. Some parts of the plants, fruits and vegetables that are produced are not redeemed edible and are considered waste. This waste is usually poorly managed, being an enormous pollutant of the planet. Therefore, finding a way to treat, reutilize or properly dispose of this waste is of utter importance [10]. In this sense, by integrating plant extracts into cosmetic formulations, an additional life-cycle step is introduced, resulting in a reduced environmental impact and fostering a more sustainable approach to minimize harm to the environment. One example of an agricultural by-product is onion peel, which is rich in bio-compounds that are characterized by their photoprotection capacity [7].

Onion (*Allium cepa*) peel is derived from one of the most popularly consumed vegetables worldwide. Onions’ production is increasing due to their medicinal potential, functional properties, and nutritional value. Annually, their production has been surpassing 90 million tons, reaching 106.59 million tons in 2021. However, nearly 600 million tons of waste are produced consisting of the onions’ peels and roots and damaged onions. Due to their inability to be used for animal feed or not being properly disposed of, the amount of waste generated is causing environmental stress and methods to reuse them are a need [11]. Onion peel presents a valuable by-product that holds diverse potential applications due to its composition of bioactive compounds, notably alk(en)yl cysteine sulfoxides, flavonoids (such as quercetin and kaempferol), flavanols and tannins [11,12]. Kumar, Barbhai [12] also refers to the presence of stilbenes (namely, resveratrol) in ethanolic extractions. Alk(en)yl cysteine sulfoxides are responsible for the flavor of the bulb, while flavonoids determine the color of both the peels and the bulb. The dried peel of onion is a great source of flavonoids (if yellowish) and anthocyanins (in red varieties) [13].

Onion peels are considered inedible and have been less studied and chemically characterized than the edible bulb. Nonetheless, a few studies have classified onion skin as a product with great commercial interest due to its content of flavonoids and other polyphenols, both categories of phenolic compounds [13].

Phenolic compounds are plants’ secondary metabolites and act as a protection for the plant [14]. These compounds possess an extensive range of biological activities due to the different structures they can present, being able to act as antioxidants and anti-inflammatory, antiallergenic, antimicrobial, and photoprotective agents, among others [14,15].

One of the most important class of polyphenols regarding their photoprotection capacity is flavonoids due to their both absorbing UV radiation and reducing ROS (reactive oxygen species) oxidative stress. They are able to absorb UV radiation due to the double bonds present in their structure. Their ability to reduce ROS oxidative stress comes from the hydroxyl groups that are connected to the aromatic rings [16]. Vanillic acid, caffeic acid, rosmaniric acid and resveratrol are among the non-flavonoid phenolic compounds that have been reported to have photoprotection capacity [16]. Dunaway, Odin [17] performed in vivo studies that showed significant inhibition of edema and inflammation with topical application of resveratrol on mice prior to their exposure to UVB radiation [18].

However, phenolic compounds are easily oxidized, losing their bioactive value, which creates the necessity to find methods to protect them, such as microencapsulation [19].

Encapsulation is defined as a technique in which an encapsulating agent (wall or shell) packs a solid, liquid, or gaseous substance (active core material) to release particles with a specific geometry at the nanometer, micrometer, or millimeter scale [20,21].

To achieve microparticles with the desired properties is crucial in choosing the most adequate coat material and encapsulation method [21,22,23].

The main phenolic compounds present in onion peel extracts have been successfully microencapsulated by molecular inclusion using β-cyclodextrin as the encapsulating agent [24,25].

Cyclodextrins (CDs) are often applied in the pharmaceutical and food industry as drug carriers to improve solubility, stability, and bioavailability of the bioactive compounds [26]. They are approved by the Food and Drug Administration (FDA) and are classified as friendly to the human body. CDs result from the degradation of starch. They are saccharides (with 6, 7, 8 glucose residues) and appear, in nature, in the α-, β- and γ-forms. Their molecules present a cone shape/format; the inner part of the cone is hydrophobic and the outer surface is hydrophilic. This characteristic confers them the ability to form inclusion complexes with poorly water-soluble molecules, such as polyphenols [25].

The most commonly used is β-CD, as it is the least expensive. β-CD-based molecular inclusion complexes are systems that trap a molecule (core material) into the cavity of the β-CDs by hydrogen bonds, van der Waals forces, or due to the hydrophobic effect [27].

Microencapsulation technology has been employed to protect the bioactive compounds present in onion peel (OP) extract and enhance its industrial applications [28,29,30]. However, to the best of our knowledge, there have been no previous studies examining the encapsulation of OP extract by molecular inclusion using β-CD as the wall material.

In this sense, this study aimed to conduct the extraction, identification, and quantification of bioactive compounds present in OP extract and to determine the sun protection factor of the obtained OP extract. Furthermore, the bioactive compounds of the OP extract were microencapsulated and characterized to determine the particle size distribution, encapsulation efficiency, antioxidant capacity, morphology, thermal properties, and stability. Subsequently, these microencapsulated compounds were incorporated into sunscreen formulations, allowing for a comprehensive comparison and evaluation of their performance as a natural UV filter in cosmetic products by determining the photoprotection evaluation of skin reactivity.

## 2. Results and Discussion

### 2.1. Onion Peel Extract Characterization

The present study aimed to compare the performance of free OP extract to the performance of microencapsulated OP extract as natural UV filters to reduce the utilization of synthetic UV filters. OP extract was obtained by solid–liquid extraction and was characterized regarding its biological properties. The results regarding the antioxidant capacity and the quantification of the main phenolic compounds of the onion peel extract are presented in Table 1.

It is important to note that the TPC (total phenolic content) value obtained is within the range of the literature values. Nevertheless, it is closer to the higher values, demonstrating a high phenolic content in this extract [30,31,32]. Furthermore, the antioxidant capacity of the OP extract was also evaluated by the DPPH assay and ABTS assay. The obtained results appear in the range of the literature; however, they are slightly lower than those obtained for the same extraction method [7]. The results of these analyses depend on the extraction method, the solvent used, and the variety of onion used, which may lead to significant variations in the results obtained among authors. Additionally, it is critical to note that the value of the half-maximal inhibitory concentration (IC_50_), which represents the concentration of the extract needed to inhibit 50% of the DPPH radical present in the solution, is inferior to 50 mg L^−1^, which means this extract is considered a very strong antioxidant [33].

The main phenolic compounds identified by HPLC-DAD were quercetin and resveratrol, which is in accordance with the literature, although present in smaller concentrations. The literature also refers to the presence of kaempferol; however, this compound was not detected in the studied OP extract [7].

**Figure 1:**
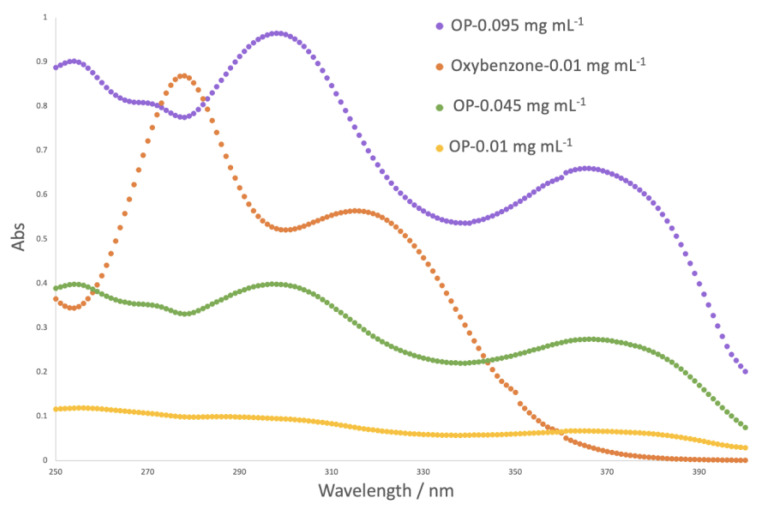
Absorbance spectra of solutions of oxybenzone and onion peel (OP) extract at different concentrations.

The sun protection factor (SPF) is a numerical indicator of a sunscreen’s protection level against UV rays that damage the skin. The higher the SPF of a product, the more effective it is at preventing sun damage. The sun protection factor was analyzed for different concentrations of onion peel (OP) extract and was compared to a synthetic UV filter serving as positive control (oxybenzone) (Table 2). The absorbance spectra are illustrated in Figure 1.

For the same concentration, the protection factor for the OP extract solution was inferior to the one obtained for the positive control. However, it is important to note that the maximum concentration of synthetic UV filter is legislated, which does not happen for the phenolic extract. The phenolic extract is a mixture of compounds, and not all contain photoprotective capacity. In order to achieve the same level of protection, a higher concentration of OP extract is needed. For higher concentrations, the results reveal higher values of SPF, demonstrating the high potential of the OP extract to be used as a natural UV filter. Figure 1 also demonstrates that OP extract might potentially provide protection against a broader range of UV radiation, as it absorbs radiation between 350 and 390 nm, which oxybenzone (synthetic UV filter) does not absorb.

### 2.2. Microencapsulation

OP extract was microencapsulated by molecular inclusion, using β-cyclodextrin as the coating agent. The two most important aspects when microencapsulating bioactive compounds are particle size and encapsulation efficiency [21].

Particles with diameters within nanometers have a higher tendency to aggregate due to the type of interaction established and to their high surface area. Furthermore, nanoparticles might be able to infiltrate the blood and cause cytotoxicity [5]. Particle size also influences the release of the core material. Materials entrapped in smaller particles have enhanced accessibility to the external phase, which might result in a faster release by diffusion, a lower drug loading, and water might penetrate the particle more easily. On the other hand, smaller particles might better adhere to the skin due to better binding per unit of particle mass compared to the binding presented for larger particles [34].

Encapsulation efficiency measures the amount of extract that was successfully entrapped in the microcapsule, and so the highest encapsulation efficiency possible is desired.

To achieve microparticles that contain the desired characteristics for cosmetic applications, the produced microparticles were optimized regarding these two factors.

**Figure 2:**
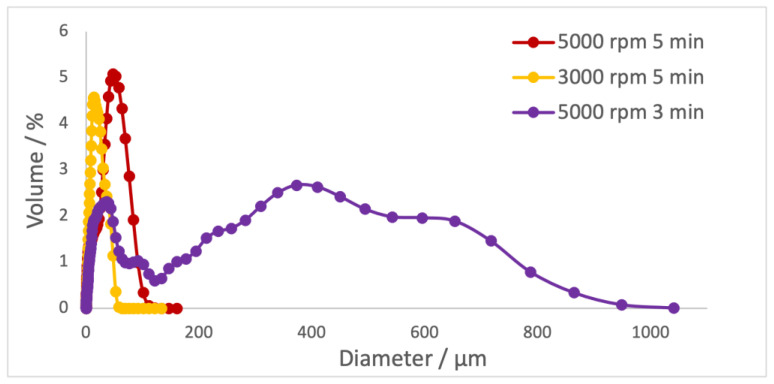
Particle size distributions of different microparticle samples.

Firstly, the effect of the rotation velocity and time of rotation on the size of the microparticles was evaluated (Table 3 and Figure 2).

Decreasing the rotation speed leads to a decrease in the particle size. Reducing the rotation time reveals an increase in the diameter of the particles and an increase in the polydispersity index. The optimum outcome would be particles with a larger diameter and a small polydispersity (5000 rpm/5 min) to guarantee a homogeneous distribution of the extract in microparticles and minimize the risk of microparticles infiltrating the blood and causing cytotoxicity.

**Figure 3:**
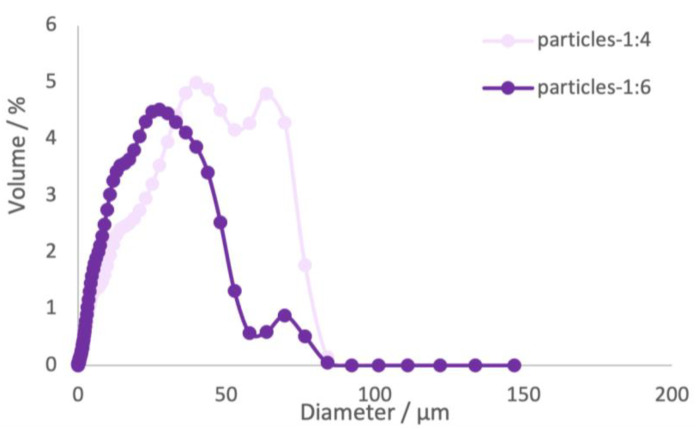
Particle size distributions of different microparticle samples.

Furthermore, the effect of the loading capacity in the microparticles was studied. The loading capacity is the amount of encapsulated material per weight unit of microparticle. Therefore, the microencapsulation protocol was followed for different ratios of mass of extract/mass of β-cyclodextrin, 1:4, 1:6, and 1:10 (*w*/*w*). The size of the microparticles and the encapsulation efficiency were evaluated for each sample (Table 4 and Figure 3).

The sample at the ratio 1:10 (*w*/*w*) did not produce a sufficient amount of sample to be analyzed by Coulter counter. Furthermore, this sample presented a low encapsulation efficiency, which led to the exclusion of this sample for further analyses and evaluations. The samples 1:4 and 1:6 exhibited a bimodal distribution of particle sizes, as illustrated in Figure 3, suggesting the formation of aggregates of microparticles during the batch production. The mean diameter values obtained for sample 1:4 was found to be higher than the values obtained for sample 1:6, measuring 31 μm and 21 μm, respectively. This difference can be associated with the higher concentration of material verified in sample 1:4. According to [35], the mean diameters of powders can be influenced by various factors, such as the nature of the wall materials, the type of core material, concentration of wall materials, stirring speed and polymer molecular weight. For samples 1:4 and 1:6, the PDI values were higher than 1. These findings indicate that the samples exhibited a wide range of particle sizes and a greater degree of heterogeneity [35,36]. The higher PDI value obtained for sample 1:6 compared to sample 1:4, suggests a greater degree of heterogeneity in the particle size distribution. This suggests that the particles in sample 1:6 vary to a larger extent in terms of their sizes, resulting in a broader size distribution compared to sample 1:4.

Encapsulation efficiency measures the amount of extract successfully entrapped in the microcapsule and is one of the most important characteristics when evaluating encapsulation processes. The encapsulation efficiency was determined as presented in Section 3.5.2. The results show that this polymer is more efficient in the encapsulation process when presenting a high loading capacity. Akdeniz, Sumnu [30] microencapsulated phenolic extracts from onion skin using maltodextrin combined with gum Arabic, casein or whey protein concentrate as coating material. They found that the encapsulation efficiency also increased with the decrease in dextrin ratio, and also when combining maltodextrin with casein. On the other hand, for the combination of maltodextrin with gum Arabic and whey protein concentrate, the encapsulation efficiency increased with the increment in maltodextrin ratio in the coating material. Their highest efficiency was for the combination of maltodextrin with casein, with the lowest amount of dextrin, and presented a value of approximately 90%, which is in concordance with the results for this study.

Ultimately, the antioxidant capacity of the microencapsulated extract was assessed and subsequently compared with that of the non-encapsulated extract (Table 5).

The results reveal that the microencapsulated extract exhibits superior antioxidant capacity compared to the non-microencapsulated extract, a finding that is consistent with the existing literature [37,38]. Moreover, the sample with the ratio of 1 g_extract_ to 4 g_coating agent_ demonstrates better antioxidant capacity than the sample with the ratio of 1 g_extract_ to 6 g_coating agent_.

**Figure 4:**
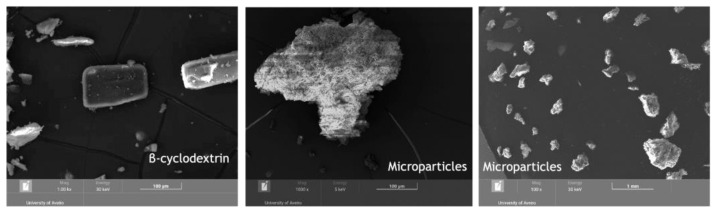
Results of the β-CD powder (coating agent) and OP extract microparticles analyzed by scanning electron microscopy (SEM).

After optimizing the protocol to obtain the desired microparticles, OP extract microparticles and the β-CD powder (coating agent) were analyzed by scanning electron microscopy (SEM) in order to examine their morphology (Figure 4).

The results revealed that the microparticles present an irregular shape, with some similarities with the β-CD powder morphology, in accordance with findings by several other authors for microparticles produced using β-CD powder as the coating agent [39,40]. The results also demonstrated that microparticles are porous. Porosity may indicate that one of the phenomena that allows the release of the OP extract is passing through the pores.

**Figure 5:**
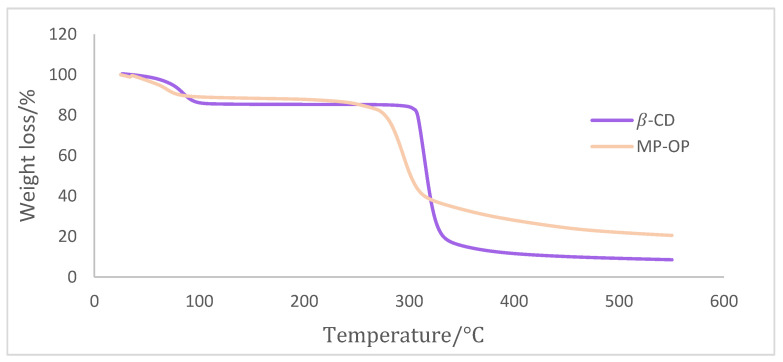
TGA analysis of the OP extract microparticles (MP-OP) and β-cyclodextrin (β-CD) powder.

In order to evaluate the thermal stability of the microparticles, two analyses were performed: thermogravimetric analysis (TGA) and differential scanning calorimetry (DSC). TGA and DSC have been considered effective tests to study alterations in physical and chemical properties of the material caused by temperature [41]. TGA analysis was performed in both β-CD powder and microparticles containing the OP extract (Figure 5).

TGA analysis revealed two thermal events of mass loss. The first event, in the range of 20 and 105 °C, is due to the evaporation of adsorbed water on the microparticles’ surface. The second event from 250 to 400 °C corresponds to the decomposition and depolymerization of the microparticle constituents. MP-OP exhibits a lower degree of weight loss at 100 °C, suggesting that it possesses a lower moisture content than β-CD.

Moreover, from 105 to 300 °C, the findings indicate that MP-OP shows lower thermal stability when compared to β-CD. Consequently, caution should be exercised when using microparticles in applications or processes that involve temperatures exceeding this threshold.

**Figure 6:**
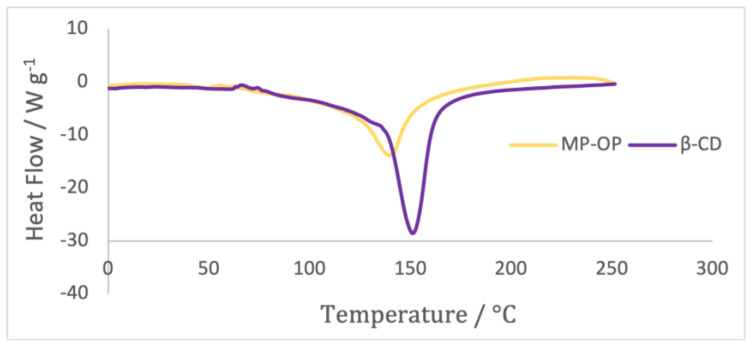
DSC analysis of the OP extract microparticles (MP-OP) and β-cyclodextrin (β-CD) powder.

Differential scanning calorimetry (DSC) is a widely employed and routine method for confirming the formation of complexes in the solid state [42]. DSC analysis was performed in both β-CD powder and microparticles containing the OP extract (Figure 6).

MP-OP and β-CD exhibited a broad endothermic event with a peak observed at approximately 140 °C for MP-OP and around 151 °C for β-CD. The endothermic peak of MP-OP shifted left by several degrees compared to β-CD, presumably due to the interaction of OP extract with β-CD molecules.

The shift or disappearance of the endothermic melting peak observed on a DSC spectrum for a guest molecule typically indicates successful encapsulation or combination [43]. Therefore, the obtained results provide evidence of successful encapsulation of onion peel extract within β-cyclodextrin.

In order to evaluate the performance as a photoprotection agent of the microencapsulated extract and compare it with the performance of the non-encapsulated OP extract, the SPF value was calculated (Table 6).

Microparticles are characterized by having a controlled release of the trapped compound; in this case, there is a controlled release of the OP extract. It is expected that, for solutions of the same concentration of OP, the solution of the microencapsulation extract presents a smaller amount of free extract, as a significant amount should still be entrapped at the time of the analysis. A smaller amount of free extract should lead to an inferior protection factor, as demonstrated by the results.

### 2.3. Sunscreen Formulations

**Figure 7:**
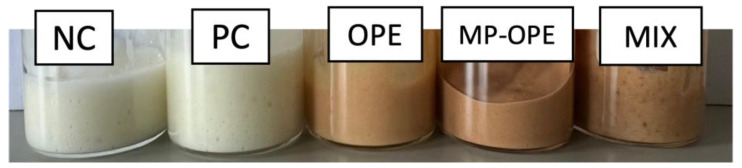
Sunscreen formulations produced for this study. NC—negative control formulation; PC—positive control formulation; OPE—onion peel extract formulation; MP-OPE—microencapsulated onion peel extract formulation; MIX—mixture formulation.

Five oil-in-water formulations were produced (Figure 7). As a negative control, a formulation without additives was made. Synthetic UV filter oxybenzone was added at a concentration of 5% to the positive control (PC) formulation, and it was chosen since it is one of the most widely used synthetic UV filters in commercial sunscreens [44]. To study the effect of OP extract in the formulations and compare its photoprotective performance to the performance of the synthetic UV filter, a formulation containing 5% of OP extract (OPE), non-encapsulated, was produced. In order to evaluate microencapsulation as a protection technique, a formulation containing 5% of microencapsulated OP extract (MP-OPE) was also developed. Finally, a formulation (MIX) containing a mixture of 1.6% of each of the three additives (oxybenzone, non-encapsulated, and microencapsulated OP extract) was produced to perform a more thorough evaluation and comparison.

The stability of the formulations was evaluated over a 5-week time span. All formulations maintained organoleptic qualities, such as color and smell, unaltered over the course of the study. NC and PC formulations had a beige color, while the other formulations presented a brownish color due to the presence of the OP extract. MP-OPE and MIX formulations were darker than the OPE formulation since it was necessary to use a larger amount of microparticles (also brownish) to achieve the same percentage of OP extract used in OPE formulation. In terms of aroma, NC and PC were odorless, while the rest presented a light onion aroma. No creams demonstrated any changes in smell over the time of the study. Regarding their appearance/consistency, MP-OPE and MIX formulations had small visible particles, while the remaining formulations were completely homogeneous.

**Figure 8:**
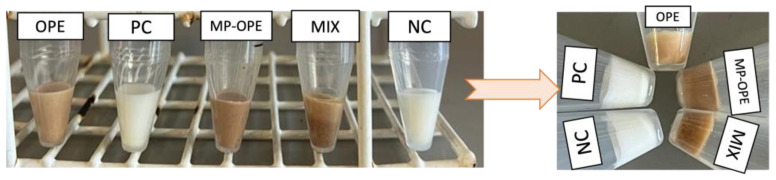
Results of the centrifuge test for the week of production of the formulations and 4 weeks after production. NC—negative control formulation; PC—positive control formulation; OPE—onion peel extract formulation; MP-OPE—microencapsulated onion peel extract formulation; MIX—mixture formulation.

To evaluate the formulations’ physical stability, a centrifuge assay was performed (Figure 8). The centrifuge test revealed that none of the formulations presented phase separation, confirming the stability of the emulsions. Nevertheless, the undissolved granules were deposited at the bottom of the MIX formulation falcon.

The formulations were also subjected to a thermal stability test to simulate the long-term shelf life with temperature changes between 4 and 50 °C. The results revealed no changes in color, texture, smell, or mass, indicating the emulsions presented high long-term stability and maintained their properties regardless of temperature changes.

**Figure 9:**
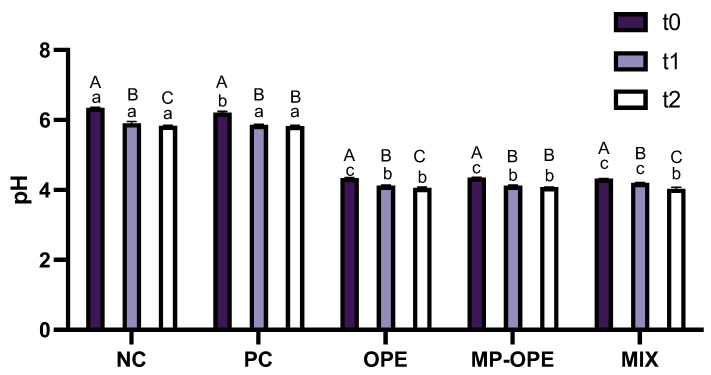
pH values for the sunscreen formulations at the 3 analysis times. NC—negative control formulation; PC—positive control formulation; OPE—onion peel extract formulation; MP-OPE—microencapsulated onion peel extract formulation; MIX—mixture formulation. Different lowercase letters (a–c) in the same column represent statistically different values (*p* < 0.05) between the formulations for each analysis time. Different capital letters (A–C) in the same line represent statistically different values (*p* < 0.05) between the two analysis times for each formulation. t0—2 days after production; t1—two weeks after production; t2—four weeks after production.

Skin pH is within the range of 4 to 6, and any cosmetic formulation produced with the intent of topical application should present pH values within this range [45]. The pH of the formulations was determined for three different analysis times: week of production, two weeks after production and four weeks after production (Figure 9).

Results reveal that the pH of all formulations slightly decreases over time. Nevertheless, all formulations’ pH values remain within the ideal range for topical application. Furthermore, it is demonstrated that the presence of OP extract in the formulations acidifies them, which is expected since phenolic compounds are weak acids [7].

**Figure 10:**
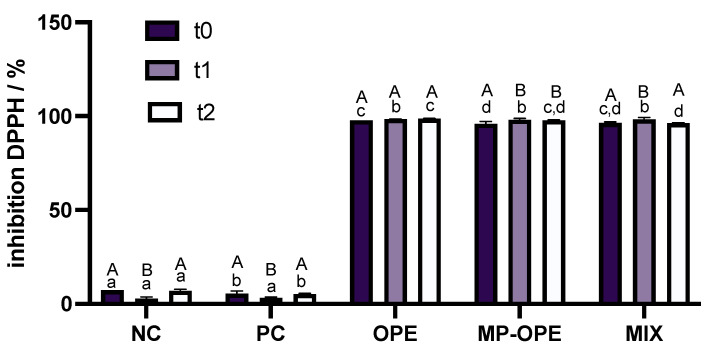
Percentage of DPPH inhibition for the five formulations at the 3 analysis times. NC—negative control formulation; PC—positive control formulation; OPE—onion peel extract formulation; MP-OPE—microencapsulated onion peel extract formulation; MIX—mixture formulation. Different lowercase letters (a–d) in the same column represent statistically different values (*p* < 0.05) between the formulations for each analysis time. Different capital letters (A, B) in the same line represent statistically different values (*p* < 0.05) between the two analysis times for each formulation. t0—week of production; t1—two weeks after production; t2—four weeks after production.

Antioxidants react with free radicals, neutralizing these and preventing the oxidation of the formulations [46]. Synthetic antioxidants are present in cosmetic formulations in low concentrations, for example, butylated hydroxytoluene (BHT) is used in a concentration of 0.50% [47]. To study OP extract as an antioxidant, it would be necessary to produce a formulation with 0.50% of OP extract and compare it with a positive control formulation containing a synthetic antioxidant. However, the scope of this study was to evaluate the behavior of OP extract as an alternative UV filter and not as an antioxidant, and so no formulation containing only 0.50% of OP extract was produced. Nevertheless, the antioxidant capacity of the formulations was evaluated by the DPPH assay, in order to elucidate the behavior of phenolic extract in formulations (Figure 10).

The results reveal that neither NC nor PC present antioxidant capacity, while the formulations containing the phenolic extract are able to inhibit almost completely the DPPH radical, presenting very strong antioxidant capacity. These observations are in accordance with the anticipated results, since NC and PC contain no antioxidants in their composition, and hence no oxidation was prevented. Phenolic compounds are known for their antioxidant capacity [14], and formulations containing the extract were expected to be able to postpone and reduce oxidation, which was demonstrated by the results.

Synthetic antioxidants’ concentration on cosmetic formulations are limited by legislation. As previously stated, agricultural by-product extracts are constituted by several other bioactive compounds, such as vitamins, which allows their use in higher concentrations, increasing the antioxidant power of the formulations.

The sun protection factor (SPF) was calculated to evaluate the photoprotection capacity of the formulations over time (Table 7).

The results reveal that NC presents no protection factor against UV radiation, which was expected since no additive/UV filter/extract was added to this formulation. PC formulation presented the highest value of SPF.

In terms of the formulations containing OP extract, the analysis is more complex. In the week of production of the sunscreens, the formulation containing microencapsulated extract (MP-OPE) presented a higher protection factor than both the OPE and MIX formulations. For the second analysis time, two weeks after production, and for the final analysis time, four weeks after production, MIX emulsion presents the highest SPF value. OPE formulation always presents the lowest protection factor, which may be justified by the fast degradation of the OP extract while not protected. Considering the variation in the protection factor, it is possible to further discuss the results presented above. The results reveal that between t0 and t2, the MP-OPE formulation presents a constant decrease in the SPF value, which may be justified by the controlled release of the OP extract when microencapsulated. Extract release from the microparticles is expected to peak in the first few hours, followed by a constant release of the extract from that point on. Considering that the first analysis was performed two days after production of the cream (t0), the microparticles in the formulation should be releasing OP extract at a constant rate, leading to a proportional decrease in the SPF value with time. On the other hand, the OPE formulation contains no encapsulated extract, leading to a greater decline in the value of the SPF between the first two analyses. At t1 (two weeks after production), the majority of the OP extract had already been degraded. The existence of a smaller amount of extract in the formulation between t1 and t2 (four weeks after production) leads to a less abrupt decrease in the protection factor value.

These results reveal that microencapsulation protects the extract from degradation. When incorporated in cosmetic formulations, the microencapsulated extract enables sunscreens to maintain their photoprotection for longer periods, increasing sunscreen shelf life. MIX and PC formulations demonstrated the best results, presenting almost no decline in the SPF value. The results suggest that the OP extract, either free or microencapsulated, presents itself as a prospective natural UV filter to substitute synthetic UV filters or combined with these to decrease the use and concentration of the latter.

**Figure 11:**
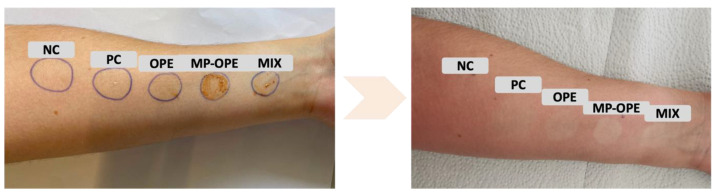
Volunteer’s skin reaction before and after 1 h of sunlight exposure. The first photograph was taken right before sun exposure, and the second was taken 3 h after the skin being exposed to 1 h of sunlight. NC—negative control formulation; PC—positive control formulation; OPE—onion peel extract formulation; MP-OPE—microencapsulated onion peel extract formulation; MIX—mixture formulation. Skin reaction test was performed at 4 weeks.

Finally, the photoprotection capacity of the formulations was further evaluated by studying a volunteer’s skin reactivity to the formulations after one hour of sunlight exposure (Figure 11). The volunteer had healthy skin.

As expected, the skin area to which the NC formulation was applied displayed the greatest irritation, as this formulation had no additive to protect against UV radiation. All the other formulations demonstrated protection against UV radiation, which is in concordance with the results obtained by the SPF value test. Visually, it appears that formulations containing OP extract provide higher protection. MP-OPE and MIX formulations presented the best results, with the skin area where they were applied appearing less tanned and irritated than the rest. Figure 11 shows that the OPE formulation presents higher protection than the PC, but protects less against UV radiation than the MP-OPE and MIX formulations. The results reveal that by microencapsulating the extracts, protection of the OP extract is successfully accomplished, preserving the photoprotection capacity of the extract and enhancing its protection against UV radiation.

The results of this test appear to contrast with the obtained SPF values. However, SPF only evaluates photoprotection in the UVB region. OP extract is a mixture of compounds that, due to their synergy, absorb UV radiation in other regions of the UV radiation spectrum, providing higher protection than what the SPF value reveals.

Even though these are preliminary studies, they suggest that OP extract, either non-encapsulated or microencapsulated, might be used as a source of photoprotection for more sustainable and environmentally friendly sunscreens.

## 3. Materials and Methods

### 3.1. Samples and Reagents

The onion peels used in this work were obtained from white Valencian onions (*Allium cepa* L.) that were cultivated in early April 2022 and harvested at the end of August 2022, in Campo Maior, Portugal. In this culture, only phosphonium (at the time of the cultivation) and urea (at half of the growth time) were used as fertilizers. Ethanol (extraction solvent) was bought from VWR (Rosny-sous-Bois, France). The reagents used for the antioxidant capacity assays, 2,2-diphenyl-1-picrylhydrazyl (DPPH), 2,2′-azino-bis(3-ethylbenzothiazoline-6-sulfonic acid) (ABTS) and Folin–Ciocâlteu reagent, were obtained from Sigma Aldrich (St. Louis, MO, USA). In the HPLC analysis, the quercetin standard and resveratrol purchased from Sigma Aldrich (St. Louis, MO, USA) was used; the solvents consisted of ethanol, ultrapure water, and acetonitrile purchased from Sigma Aldrich (St. Louis, MO, USA). For the sun protection factor calculation, benzophenone-3 (oxybenzone) was also purchased from Sigma Aldrich (St. Louis, MO, USA). For the microencapsulated process, the β-cyclodextrin utilized was obtained from Sigma Aldrich (St. Louis, MO, USA) and ultrapure water as a solvent. Methanol purchased in Carlo Erba (Barcelona, Spain) was used in the assays to characterize the microparticles. For sunscreen formulations, products utilized were glycerine (Ref. COSM-01216), xanthan gum, sweet almond oil, and betaine purchased from GranVelada (Zaragoza, Spain) and lecithin acquired from TCI (Tokyo, Japan).

### 3.2. Extraction of the Phenolic Content

Firstly, OP was dried in a drying oven (P. Selecta, Mod. 210, N. 16472) at 70 °C for 17 h, and then milled to a diameter inferior to 1 mm using a coffee grinder (Q 5321 Qilive, Auchan, Croix, France). A solid–liquid extraction with a Soxhlet apparatus was utilized to obtain the OP extract, according to a modified protocol [48]. The solvent used was ethanol, in a mass-solvent ratio of 1:20 m/V. The extraction was performed for 2 h at a temperature of 70 °C and in triplicate. The solvent was later evaporated by a rotary evaporator (Rotavapor R-200, BUCHI Laboratories, Flawil, Switzerland) coupled to a vacuum pump CVC 3000, with a bath temperature of 34 °C and the pressuring varying from 100 to 45 mbar. Finally, the extracts were subjected to a gentle stream of nitrogen to guarantee that all the solvent had evaporated.

### 3.3. Characterization of the Phenolic Extract

#### 3.3.1. Total Phenolic Content

The Folin–Ciocâlteu method was utilized to determine the total phenolic content (TPC) of the OP extract. To proceed with the analysis, it followed an adapted protocol from the literature [49]. Briefly, 20 μL of the sample was added to a 2 mL cuvette, along with 100 μL of Folin–Ciocâlteu reagent and 1580 μL of water. After 3 to 6 min, 300 μL of sodium carbonate solution with a concentration of 333.3 g L^−1^ (saturated solution) was added to the cuvette, and the resulting solution was incubated for 2 h in the dark at room temperature. After the incubation period, the absorbance of the solution was determined using a Thermo GENESYS 10S UV-Vis spectrophotometer at a wavelength of 750 nm. The results were expressed in gallic acid equivalents (GAE).

#### 3.3.2. DPPH and ABTS Assays

The antioxidant activity of the OP extract was evaluated by 2,2-diphenyl-1-picrylhydrazyl (DPPH) and 2,2-azino-bis(3-ethylbenzothiazoline-6-sulfonic acid) (ABTS) assays.

When performing the DPPH assay [50], 20 μL of the sample/Trolox standard was added to 180 μL of DPPH solution, at a concentration of 150 μmol L^−1^, and left to incubate in the dark for 40 min. Then, the absorbance was read at 515 nm in the computer software Gen5 (Agilent Bio Tek, Santa Clara, CA, USA). As a blank, a solution of 20 μL of water and 180 μL of ethanol was utilized, and as a control, 20 μL of water was added to 180 μL of DPPH solution. The percentage of DPPH inhibition was calculated through Equation (1), where Abs_sample_ refers to the absorbance of the control, Abs_blank_ refers to the absorbance of the blank and Abs_control_ is the measured absorbance for the sample solutions.

DPPH inhibition % = (1 − ((Abs_sample_ − Abs_blank_)/(Abs_control_ − Abs_blank_))) × 100, (1)

The results were expressed in Trolox equivalent (TE). Hence, a Trolox calibration curve, based on the percentage of DPPH inhibition of Trolox standards, was prepared with a concentration between 25 and 250 mg L^−1^. The extract concentration necessary to inhibit 50% of DPPH (IC_50_) was also calculated.

The ABTS assay was carried out following the [51] protocol. To perform the ABTS assay, OP extract solutions were prepared in ethanol with the concentration varying from 20 to 150 mg L^−1^; 20 μL of these solutions, or the Trolox standard solutions, were added to 180 μL of ABTS solution and were then incubated in the dark for 15 min. In the end, the absorbance was read at 734 nm in the computer software Gen5 (Agilent Bio Tek). As a control, a solution of 20 μL of 0.05 M acetic acid buffer solution (pH 4.6) and 180 μL of ABTS solution was utilized.

The percentage of ABTS inhibition was calculated using Equation (2), where Abs_control_ refers to the absorbance of the control and Abs_sample_ is the measured absorbance for the sample solutions. To express the results in TEAC, a Trolox calibration curve was prepared.

ABTS inhibition % = ((Abs_control_ − Abs_sample_)/Abs_control_) × 100 (2)

#### 3.3.3. Identification and Quantification of Phenolic Compounds in the OP Extract by HPLC-DAD

The phenolic compounds present in the OP extract were quantified by high-performance liquid chromatography with a diode array detector (HPLC-DAD). The analyses were performed using an Elite LaChrom (Hitachi, Tokyo, Japan) HPLC system equipped with a Hitachi L-2200 autosampler L-2130 pump and an L-2455 diode array detector. The solvent used to dissolve the samples was a mixture of acetonitrile, water and ethanol in a ratio of 2:1:1 *v*/*v*/*v*. Samples were injected in a Puroshper STAR RP-18 LiChroCART chromatography column (Merck, Darmstadt, Germany), and phase A was composed of ultrapure water with 0.5% of orthophosphoric acid, while phase B consisted of methanol:acetonitrile (80:20 *v*/*v*).

The phenolic compounds were quantified by the external standard method [7] as presented in the Supplementary Material. Quercetin was quantified following Equation (S1), using the areas measured in Figures S1 and S2. Resveratrol was quantified following Equation (S2), using the areas measured in Figures S3 and S4.

#### 3.3.4. Sun Protection Factor

The photoprotection capacity of the onion peel extract was determined by calculating the sun protecting factor (SPF) following a modified protocol [52].

Firstly, ethanolic solutions of OP extract, with a concentration of 0.01, 0.045 and 0.095 mg mL^−1^, were prepared. A 0.01 mg mL^−1^ solution of (2-Hydroxy-4-methoxyphenyl)-phenylmethanone (oxybenzone) was also prepared to be used as a positive control for a comparison between the performance of the OP extract with a synthetic UV filter. Oxybenzone was chosen as a positive control, as it is one of the most used synthetic UV filters in commercial sunscreens.

The absorptions values of the samples were measured in a UV-3100PC spectrophotometer (VWR, Darmstadt, Germany) in the wavelength range of 280 to 400 nm. The SPF values were calculated using Equation (3), in which CF is the correction factor equal to 10, EE(λ) refers to the erythemal effect spectrum, I(λ) is the solar intensity spectrum for a specific wavelength λ, and EE(λ) × I(λ) represents the normalized product function. (λ) refers to the absorbance value of the sample at the wavelength λ.

(3) $$\mathrm{SPF}=\mathrm{CF}\;\times \;\sum_{290}^{320}\mathrm{EE}\left(\lambda \right)\;\times \;I(\lambda )\;\times \;\mathrm{Abs}\left(\lambda \right)$$

### 3.4. Microencapsulation

The microencapsulation of the OP extract was performed according to a modified protocol [53]. Firstly, OP extract and β-CD (beta-cyclodextrin) were solubilized in 250 mL water, using the ratio of 1 g of extract to 4 g of β-CD. The solution was then put in an ultrasound bath for 5 min, followed by a period of 2 h of magnetic stirring at 1000 rpm. Furthermore, the solution was subjected to a high-performance homogenizer, Ultra-Turax (IKA T18 Digital ULTRA-TURAX), for 5 min at a velocity of 5000 rpm. Finally, the obtained solution was vacuum filtered using 0.20 μm PVDF (polyvinylidene difluoride) filters.

The filters containing the microparticles were frozen at −80 °C for 18 h and then freeze-dried for 24 h to ensure no water was present in the microcapsules. The resulting microparticles were stored in a desiccator at room temperature and covered with paper foil to protect them from light.

### 3.5. Characterization of the Microparticles

#### 3.5.1. Particle Size Distribution

The particle size distribution and polydispersity were performed using a Coulter counter LS 230 particle size analyzer (Miami, FL, USA) equipment. The analyses were performed in triplicate, and the results presented are the average values.

The polydispersity of particle size distributions was evaluated according to the polydispersity index (PDI) using Equation (4) [54].

PDI = ((D_v,90_ − D_v,10_)/D_v,50_), (4)

$D_{v,90}$ represents the maximum particle diameter below which 90% of the sample volume exists, $D_{v.10}$ is the maximum particle diameter below which 10% of the sample volume exists, and $D_{v,50}$ consists of the maximum particle diameter below which 50% of the sample volume exists.

#### 3.5.2. Encapsulation Efficiency

To calculate the encapsulation efficiency (EE), the microcapsules were destroyed to release the encapsulated extract, which was accomplished by following a modified protocol [55]. A solution of 10 mg of powder was prepared in 4 mL of methanol and was put in an ultrasound bath for 5 min, followed by 10 min of centrifugation with an acceleration of 1502× *g*. The supernatant was filtered through 0.20 μm PVDF filters, and the filtrate was subjected to a gentle stream of nitrogen to evaporate the methanol. The obtained solid was solubilized in ethanol and analyzed via spectrophotometry.

The EE is calculated according to Equation (5), where m_b_ is the initial amount of extract used to produce 10 mg of microparticles and m_a_ is the amount of extract detected in the ethanolic solution after the microparticles’ destruction.

EE % = (m_a_/m_b_) × 100, (5)

In order to obtain the mass of extract in the ethanolic solution, a calibration curve of ethanolic solutions of OP extract was prepared, with concentrations varying from 0.010 mg mL^−1^ to 0.1 mg mL, by reading their absorbance at 290 nm on the UV-vis spectrophotometer using ethanol as blank.

#### 3.5.3. Antioxidant Capacity

The antioxidant capacity was evaluated by DPPH and ABTS assays, as described in the section “Characterization of the Phenolic Extract.”

#### 3.5.4. Morphology

The morphological structures of microparticles were analyzed by scanning electron microscope (SEM) analysis using a Tescan VEGA LMS. The samples were previously coated with a gold–palladium alloy that was applied using Agar sputter coater equipment. The SEM analysis was conducted at an accelerating voltage of 30 keV with a magnification of 100×.

#### 3.5.5. Thermal Properties

The thermal properties of the microparticles were assessed using thermogravimetric analysis (TGA) and differential scanning calorimetry (DSC) with the aid of Hitachi STA300 and Shimadzu DSC-60 instruments, respectively. In the TGA analysis, each sample weighing approximately 10 mg was heated from 25 °C to 550 °C at a constant rate of 10 °C min^−1^. For the DSC analysis, the samples were placed in sealed aluminum pans and heated from 25 °C to 110 °C at a rate of 10 °C min^−1^.

### 3.6. Sunscreen Formulations

To evaluate the effect of OP extract and its behavior as a UV filter (non-encapsulated and encapsulated) in a cosmetic formulation, five different O/W emulsions were produced. The different ingredients used, as well as their concentration in the formulation, are presented in Table 8. The base formulation consisted of 2 phases: phase A (aqueous phase) containing ultrapure water, glycerin, and xanthan gum; and phase B (oily phase) made of coconut oil, sweet almond oil, lecithin and betaine. Both phases were heated to 70 °C separately and blended until the total mixture was achieved. Then, phase B was added to phase A while maintaining temperature. The final mixture was homogenized with an IKA T18 Digital ULTRA-TURAX for 2 min at 12,000 rpm. The formulations were cooled until they reached 40 °C and the additives were added to the creams, except for the negative control, while stirring to achieve homogenization.

The first formulation consisted of the base formulation and was used as a negative control (NC). A synthetic UV filter (oxybenzone) was added to the second formulation and was used as a positive control (PC). OPE (non-encapsulated) was added to the third formulation, microencapsulated OP extract (MP-OPE) was added to the fourth formulation, and finally a mixture of the 3 additives (MIX) was used in the final formulation. Regulations state that the maximum concentration of oxybenzone in a cosmetic formulation cannot surpass 10%, which was respected in all formulations [47].

### 3.7. Stability Tests

In order to ensure the stability of the formulations, the following tests were performed 3 days after production (t0), two weeks after (t1) and four weeks after production (t2). All tests, except for the thermal stability test, were performed at room temperature, following the protocols present in the literature.

#### 3.7.1. pH Test

For each formulation, 1 g was dissolved in 4.5 mL of purified water, and its pH was measured using a digital pH meter. The sample was kept under stirring for the test [7].

#### 3.7.2. Centrifuge Test

Each formulation was evaluated regarding its physical stability by being subjected to 10 min of centrifugation at 2670× *g*. Physical stability was assessed by visually verifying if phase separation and color changes occurred [7].

#### 3.7.3. Thermal Stability Test

Heat–cold–heat cycles were used to study the thermal stability of formulations. Formulations were incubated at 50 °C for 24 h, then stored at 4 °C for 24 h, followed by 24 h of incubation at 50 °C, and finally, 24 h at 4 °C to complete the cycle, based on a modified protocol [56]. Thermal stability was evaluated by mass changes and visually by verifying textural and color consistency.

### 3.8. Antioxidant Capacity

To study the effect of OP extract on the antioxidant capacity of the sunscreen, the phenolic compounds were extracted from the formulations. Two grams of sunscreen formulations was added to 4 mL of ethanol. The solution was homogenized for 1 min in a vortex, placed in an ultrasound bath for 5 min, and centrifuged at 1502× *g* for 5 min. The supernatant was collected and stored, then 4 mL of ethanol were added to the pellet and the previous cycle was repeated. The supernatants were homogenized and used to perform the DPPH assay. The DPPH assay was performed following the protocol described above.

### 3.9. Sun Protection Factor

To evaluate the performance of OP extract, free and microencapsulated, as a natural UV filter and compare its performance against a synthetic UV filter, the sun protection factor of the formulations was evaluated following the protocol presented above in the section “Characterization of the Phenolic Extract”.

### 3.10. Photoprotection Evaluation—Skin Reactivity

All the formulations were applied to the inner arm of a volunteer in a marked area. The volunteer was exposed to sunlight for one hour. The skin reaction was visually evaluated a few hours after exposure.

### 3.11. Statistical Analysis

In order to compare the obtained results, a statistical one-way analysis of variance (ANOVA) was performed by calculating the *p*-value (95% confidence), with *p*-values below 0.05 indicating no significant difference between measures.

## 4. Conclusions

The primary purpose of this study was to compare the performance of onion peel extract, free and microencapsulated, as a natural UV filter to develop a value-added sunscreen. The onion peel (OP) extract was found to have a high antioxidant capacity and significant photoprotection capacity, justified by the presence of quercetin and resveratrol, its main phenolic compounds. Microencapsulating the extract enhanced its antioxidant capacity and stability. Molecular inclusion using β-cyclodextrin as the coating agent led to microparticles with a mean diameter of 31 μm. An encapsulation efficiency of 91.8% was achieved, confirming the successful entrapment of the OP extract. The incorporation of both extracts in sunscreen formulations led to stable cream formulations that presented significant photoprotection capacity. The formulation containing microencapsulated extract presented a higher protection factor than the formulation containing free OP extract. The best results were provided by the formulation containing a mixture of additives (synthetic UV filter and OP extract, free and microencapsulated) by maintaining the high protection factor over time. Overall, the results allow us to infer that OP extract, either free or encapsulated, presents itself as a prospective natural UV filter to substitute synthetic UV filters, or combined with these, to decrease the utilization and concentration of the latter. Microencapsulating the OP extract ensured the protection of the extract’s photoprotective capacity for longer periods, increasing the sunscreen’s shelf life. Nevertheless, given the preliminary phase of the tests performed, it would be interesting to perform other analyses in the future, mainly rheology tests and controlled release studies. It would also be important to identify other compounds present in the extract to guarantee the lack of harmful compounds in the OP extract. In conclusion, OP extract, either free or microencapsulated, presents itself as a natural UV filter to replace synthetic UV filters. Furthermore, by using a by-product agricultural extract, it is possible to add a new step in the lifetime of onion peels, reducing the impact on the environment and contributing towards a circular economy.

## Figures and Tables

**Table 1:** Obtained results for the characterization of the onion peel extract.

Test		Mean ± σ
**TPC (mg_GAE_ g^−1^_extract_)**		585 ± 10
**DPPH**	TE (mg_TE_ g^−1^_extract_)	200 ± 1
	IC_50_ (mg L^−1^)	31 ± 2
**ABTS (mg_TEAC_ g^−1^_extract_)**		381 ± 8
**HPLC-DAD**	Quercetin (mg g^−1^_dried extract_)	4.48 ± 0.01
	Resveratrol (mg g^−1^_dried extract_)	0.51 ± 0.02

The results are expressed as means ± standard deviations of 4 independent measurements. TPC: total phenolic content; GAE: gallic acid equivalent; IC_50_: concentration of extract necessary to inhibit 50% of DPPH radical; TE: Trolox equivalent; TEAC: Trolox equivalent antioxidant capacity.

**Table 2:** SPF values for solutions of OP extract at different concentrations and for a solution of oxybenzone.

Sample	Concentration (mg mL^−1^)	SPF
Oxybenzone	0.010	38.84
OP extract	0.010	6.05
OP extract	0.045	24.92
OP extract	0.095	60.24

OP—onion peel; SPF—sun protection factor.

**Table 3:** Polydispersity index (PDI) and mean diameter of different microparticle samples.

Sample	PDI	Mean Diameter (μm)
5000 rpm/5 min	2.29	32.30
5000 rpm/3 min	12.1	163.1
3000 rpm/5 min	2.25	16.23

**Table 4:** Polydispersity index (PDI), mean diameter and encapsulation efficiency of different microparticle samples.

Sample (g:g)	PDI	Mean Diameter (μm)	EE %
1:4	2.09	31 ± 22 ^a^	91.81 ± 0.03 ^a^
1:6	2.23	21 ± 16 ^a^	72.03 ± 0.05 ^b^
1:10	-	-	19.71 ± 0.04 ^c^

EE—encapsulation efficiency. Different lowercase letters (a–c) in the same column represent statistically different values (*p* < 0.05) between samples.

**Table 5:** Antioxidant capacity of different microparticles samples.

Sample (g_extract_:g_coating agent_)		TE (mg_extract_ g_Trolox_^−1^)	IC_50_ (mg L^−1^)
**1:4**	Encapsulated	304 ± 59 ^a^	23.7 ± 4.1 ^a^
	Non-encapsulated	116 ± 6 ^b^	60.7 ± 3.1 ^b^
**1:6**	Encapsulated	179 ± 16 ^c^	41.0 ± 3.5 ^b^
	Non-encapsulated	129 ± 8 ^b^	56.9 ± 3.5 ^b^

The results are expressed as means ± standard deviations of 4 independent measurements. IC_50_: the concentration of extract necessary to inhibit 50% of DPPH radical; TE: Trolox equivalents. Different lowercase letters (a–c) in the same column represent statistically different values (*p* < 0.05) between samples.

**Table 6:** Sun protection factor (SPF) values for non-encapsulated (OP) and microencapsulated (MP-OPE).

Sample Concentration (mg/mL)	SPF
OP extract (0.095)	60.24
MP-OP (0.095)	36.11

**Table 7:** Sun protection factor (SPF) values for different formulations at the 3 analysis times.

Sample	SPF t0	SPF t1	SPF t2
OPE	4.7 ± 0.1 ^a,A^	2.1 ± 0.3 ^a,B^	1.4 ± 0.4 ^a,C^
MP-OPE	18.2 ± 0.5 ^b,A^	11.0 ± 0.0 ^b,B^	6.3 ± 0.0 ^b,C^
MIX	12.9 ± 0.1 ^c,A^	11.2 ± 0.4 ^b,B^	11.0 ± 0.3 ^c,B^
NC	0.8 ± 0.1 ^d,A^	0.2 ± 0.2 ^c,A,B^	0.0 ± 0.0 ^d,B^
PC	22.8 ± 0.2 ^e,A^	19.0 ± 0.1 ^d,B^	17.8 ± 0.0 ^e,C^

NC—negative control formulation; PC—positive control formulation; OPE—onion peel extract formulation; MP-OPE—microencapsulated onion peel extract formulation; MIX—mixture formulation. Different lowercase letters (a–e) in the same column represent statistically different values (*p* < 0.05) between the formulations for each analysis time. Different capital letters (A–C) in the same line represent statistically different values (*p* < 0.05) between the two analysis times for each formulation. t0—week of production; t1—two weeks after production; t2—four weeks after production.

**Table 8:** Concentration of the several ingredients used in the sunscreen formulations.

Phase	Ingredients	Function	NC (g)	PC (g)	OPE (g)	MP-OPE (g)	MIX (g)
Phase A	Water	Solvent	73.6	73.6	73.6	73.6	73.6
	Glycerin	Humectant	7.60	7.60	7.60	7.60	7.60
	Xanthan gum	Thickener	0.60	0.60	0.60	0.60	0.60
Phase B	Coconut oil	Emollient	7.60	7.60	7.60	7.60	7.60
	Sweet almond oil	Emollient	6.80	6.80	6.80	6.80	6.80
	Betaine	Emulsifier	2.70	2.70	2.70	2.70	2.70
	Lecithin	Secondary emulsifier	1.10	1.10	1.10	1.10	1.10
Additives	Oxybenzone	Synthetic UV filter	-	5.00	-	-	1.60
	OP extract	Natural antioxidant and UV filter	-	-	5.00	-	1.60
	MP-OP	Natural antioxidant and UV filter	-	-	-	5.00	1.60

NC—negative control formulation; PC—positive control formulation; OPE—onion peel extract formulation; MP-OPE—microencapsulated onion peel extract formulation; MIX—mixture formulation; OP—onion peel; MP-OP—microencapsulated onion peel extract.

## Data Availability

The data presented in this study are available on request from the corresponding author.

## Supplementary Materials

The following supporting information can be downloaded at: https://www.mdpi.com/article/10.3390/ijms242115854/s1.
