# Supplementary material for: A Comparative Study between Onion Peel Extracts, Free and Complexed with β-Cyclodextrin, as a Natural UV Filter to Cosmetic Formulations
Source: Int J Mol Sci. 2023 Nov 1;24(21):15854. doi: 10.3390/ijms242115854 (PMC10650503; doi:10.3390/ijms242115854)
Supplement: Supplementary file 1 [file ijms-24-15854-s001.zip › ijms-2674523-supplementary.pdf]

A comparative study between Onion Peel Extracts, free and complexed with β-cyclodextrin, as a natural UV filter to cosmetic formulations

Mariana A. Messias [1,2], Sara M. Ferreira [1,2], Loleny Tavares[3,4,5] and Lúcia Santos [1,2,*]

Supplementary Material

HPLC-DAD Chromatograms

Equations (S1) and (S2) exhibit the validated calibration curves obtained in the HPLC-DAD for quercetin and resveratrol, respectively, with R values of 0.9994 and 0.9999. These curves were validated by the research group [1]. The curves were calculated in the same laboratory, HPLC equipment and conditions.

$$A = 73700 \times C_{quercetin} - 268000 \quad \text{(S1)}$$

$$A = 142000 \times C_{resveratrol} + 279000 \quad \text{(S2)}$$

Where $C_{quercetin}$ (mg L-1) and $C_{resveratrol}$ (mg L-1) represent the concentration of quercetin and resveratrol in the sample, respectively and A represents the area under the peak.

Figure S1 and Figure S2 illustrate the chromatograms obtained by HPLC-DAD for OP extract and OP extract spiked with quercetin, in order to identify and quantify quercetin in the sample.
Quercetin was identified at a retention time of 49.98 min and wavelength 365 nm.

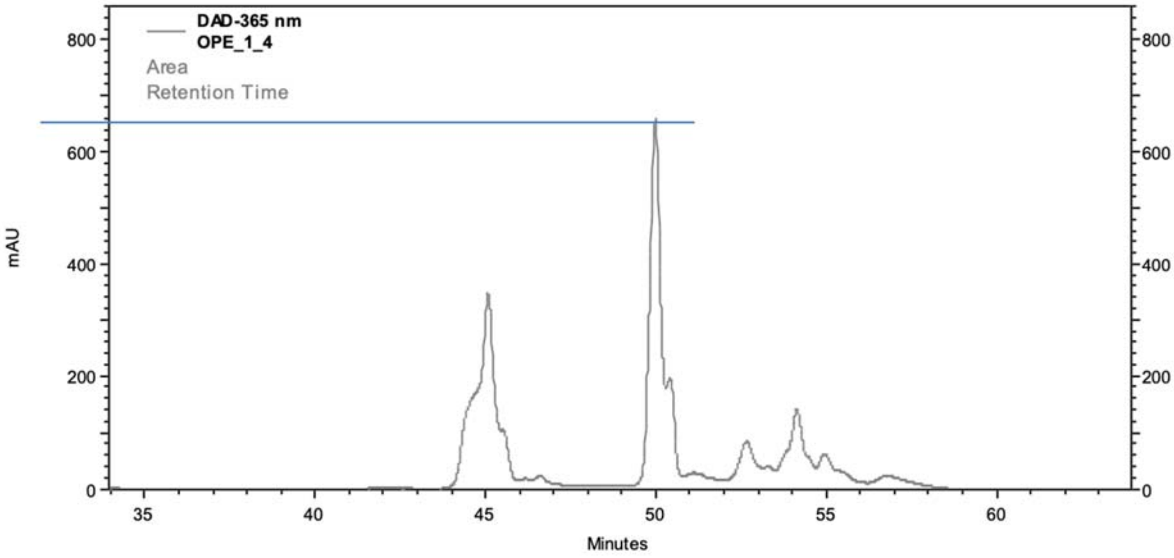

Figure S1 - Zoomed in chromatogram of the OP sample at λ = 365 nm.

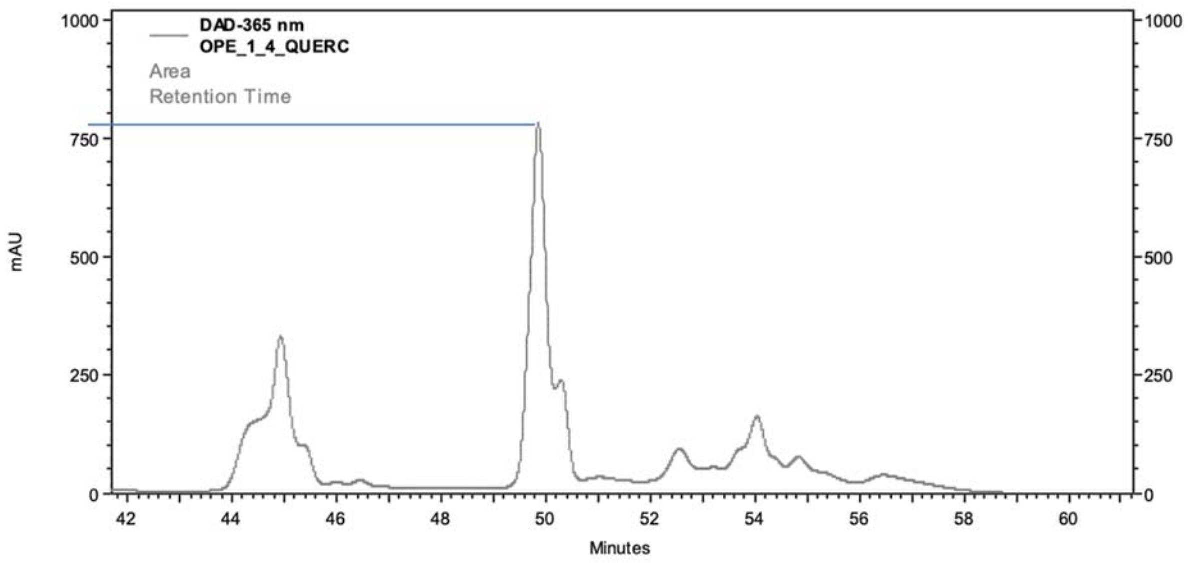

Figure S2 - Zoomed in chromatogram of the quercetin standard + OP extract sample at λ = 365 nm.

Figure S3 and Figure S4 illustrate the chromatograms obtained by HPLC-DAD for OP extract and OP extract spiked with resveratrol, in order to identify and quantify resveratrol in the sample. Resveratrol was identified at a retention time of 44.44 min and wavelength 305 nm.

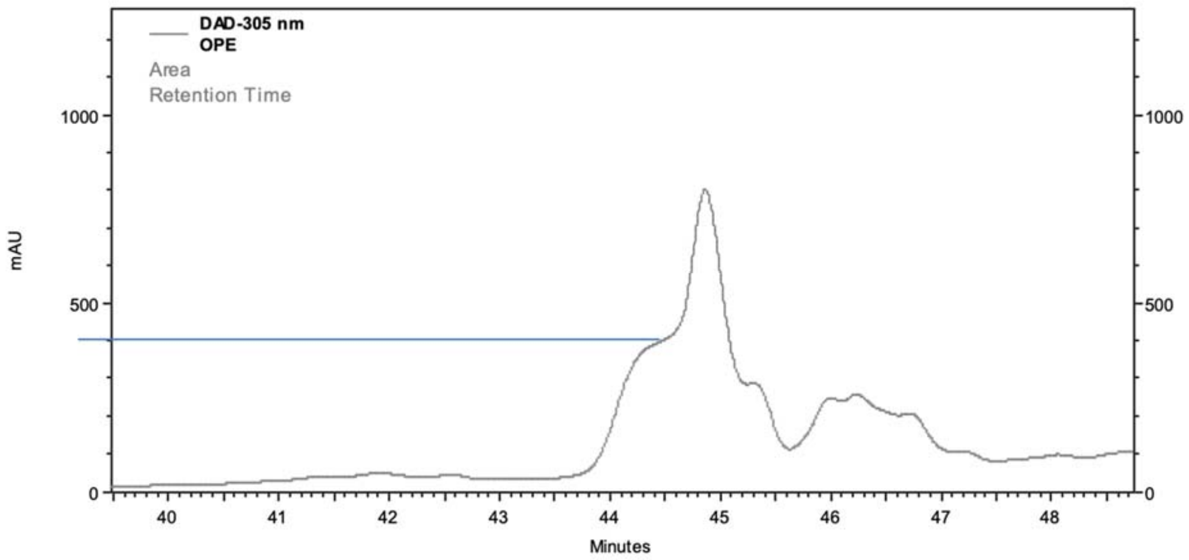

Figure S3 - Zoomed in chromatogram of the OP extract sample at λ = 305 nm.

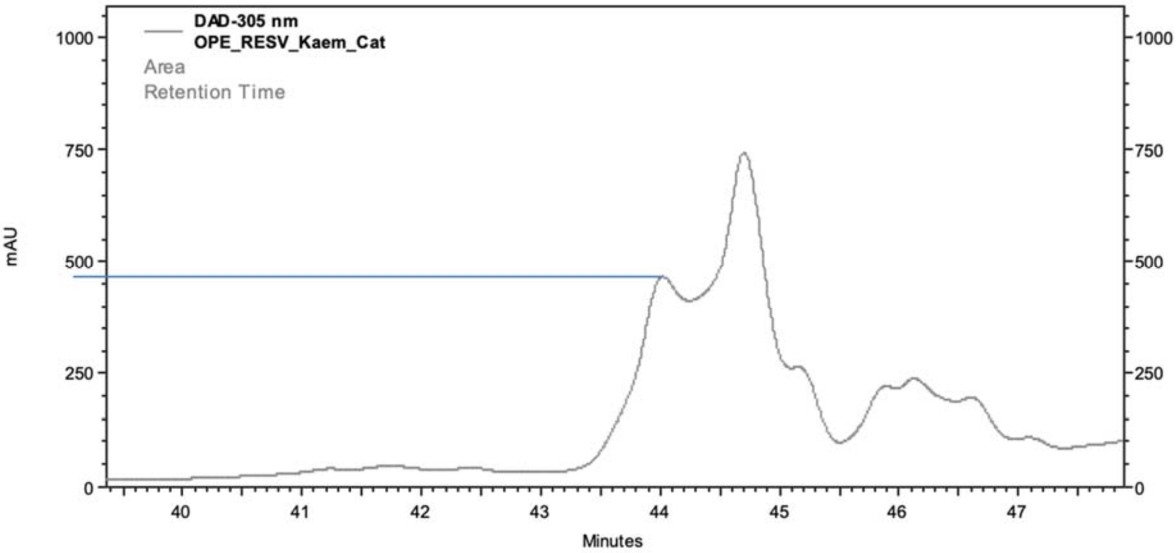

Figure S4 - Zoomed in chromatogram of resveratrol standard + OP extract sample at λ = 305 nm.
